# Eugenol Promotes Apoptosis in Leukemia Cells via Targeting the Mitochondrial Biogenesis PPRC1 Gene

**DOI:** 10.3390/cells14040260

**Published:** 2025-02-12

**Authors:** Sayer Al-Harbi, Elham M. A. Alkholiwy, Syed Osman Ali Ahmed, Mahmoud Aljurf, Reem Al-Hejailan, Abdelilah Aboussekhra

**Affiliations:** 1 Department of Molecular Oncology, King Faisal Specialist Hospital and Research Centre, P.O. Box 3354, Riyadh 11211, Saudi Arabia; 2 Department of Hematology, Stem Cell Transplant and Cellular Therapy, Cancer Center of Excellence, King Faisal Specialist Hospital and Research Centre, P.O. Box 3354, Riyadh 11211, Saudi Arabia; 3 Department of Cell Biology, King Faisal Specialist Hospital and Research Centre, P.O. Box 3354, Riyadh 11211, Saudi Arabia

**Keywords:** eugenol, apoptosis, AML, PPRC1, cancer

## Abstract

Acute myeloid leukemia (AML) is a highly heterogenous and aggressive myeloid neoplasm. To sustain growth and survival, AML cells, like other neoplasms, require energy. This process is orchestrated by mitochondria and is under the control of several genes, such as PPRC1 (PRC), a member of the PGC-1 family, which is a key player in the transcription control of mitochondrial biogenesis. We have shown here that eugenol inhibits cell growth and promotes apoptosis through the mitochondrial pathway in AML cell lines as well as in cells from AML patients but not in cells from healthy donors. Similar effects were also observed on cytarabine-resistant AML cells. Interestingly, eugenol downregulated PPRC1 at both the protein and mRNA levels and reduced mitochondrial membrane potential in AML cells. We have also shown that PPRC1 expression is higher in cancer cells from blood, breast, and other types of cancer relative to normal cells, and high PPRC1 levels correlate significantly with short overall survival (OS). In addition, PPRC1 gene mutations significantly correlate with short OS and/or disease-free survival in several cancers. PPRC1 mutations also correlated significantly with poor OS (*p* < 0.0001) when tested in a total of 23,456 cancer patients. These findings suggest an oncogenic role of PPRC1 in various types of cancer and the possible eugenol-targeting of this gene for the treatment of AML patients, especially those exhibiting resistance to cytarabine.

## 1. Introduction

Acute myeloid leukemia (AML) is the most common form of acute leukemia and is characterized by a high accumulation of hematopoietic blast cells in bone marrow due to deregulation in differentiation and proliferation signaling [1]. Clinically, only around 10% of AML patients above 60 years show five-year overall survival, while 40% of younger patients survive more than 5 years [2,3]. Despite recent advances in our understanding of AML pathogenesis, cytarabine- and idurabicin-based regimens remain the frontline treatments for AML patients. Responses of AML patients to cytarabine- and idurabicin-based therapies are often short-lived, with a median overall survival of months, emphasizing the critical need for introducing new therapeutic approaches [4,5]. Recently, a number of novel agents have been licensed for AML, but these are applicable to a limited number of patients [6,7].

Cancer cells take advantage of mitochondria/metabolism regulatory pathways to promote their tumorigenesis. The Peroxisome Proliferator-Activated Receptor γ (PPARγ) coactivator (PGC-1) family of transcriptional co-activators orchestrates mitochondrial biogenesis and energy metabolism in a variety of tissues [8]. This family consists of PGC-1α, PGC-1β, and PPRC1, which interact with several transcription factors to regulate the expression of genes involved in mitochondrial biogenesis, metabolism, and energy homeostasis [9,10]. Unlike PGC-1α and PGC-1β, the PPRC1 expression is uniquely altered by proliferation signals and is tightly regulated in a cell cycle-dependent fashion [11]. *PPRC1* knockdown showed substantial reduction in mitochondrial biogenesis, respiratory function, and cell cycle progression [12]. Mice lacking the *PPRC1* gene failed to form eggs and died after implantation, indicating a critical role of this gene in early embryonic development [13]. In fact, PPRC1 plays critical roles in linking the cell growth program to mitochondrial biogenesis [11,14]. However, the link between PPRC1 and tumor progression and/or clinical outcome of cancer patients still remains not well defined.

Eugenol [4-allyl(-2-methoxyphenol)] is a phenolic compound found in the essential oils of various plants such as *Syzgium aromaticum* (clove) and *Cinnamomum verum* (cinnamon leaf). Eugenol possesses antioxidant and anti-inflammatory effects [15]. Eugenol has also anti-cancer effects [15,16,17]. Therefore, we tested in the present study the effect of eugenol on leukemia cell lines and primary AML cells and have shown eugenol-dependent induction of apoptosis through the mitochondrial pathway and inhibition of cell proliferation in both cytarabine-sensitive and -resistant cells. Furthermore, eugenol reduced mitochondrial membrane potential and downregulated PPRC1. Using large-scale cancer transcriptome, genomics, and clinical outcome datasets, we have shown that PPRC1 plays important roles in tumor progression. Thereby, this gene could be of great therapeutic and prognostic value for various types of cancer, including leukemia.

## 2. Materials and Methods

### 2.1. Cells, Cell Culture, and Reagents

Cell lines (THP1, KG-1, K562) were obtained from ATCC (Manassas, Virginia, USA and were cultured as recommended (37 °C in a humidified incubator, 5% CO_2_). Cell lines and primary AML cells were cultured in suspension in flasks in RPMI-1640 medium supplemented with 10% fetal bovine serum and 1% antibiotic–antimycotic. All supplements were obtained from Gibco, Grand Island, NY, USA), and Cytarabine and Eugenol were purchased from Sigma-Aldrich, Saint Louis, MO, USA).

### 2.2. Blood Samples

Patients and volunteers were first consented under RAC#2190003. Blood samples (5 mL) were collected from diagnosed AML patients before treatment and healthy volunteers in sodium heparin tubes. Peripheral blood mononuclear cells (PBMCs) were isolated using Ficoll-paque plus density gradient centrifugation.

### 2.3. Cytotoxicity Assay

Cells (5 × 10^3^) were first seeded in 96-well plates with 100 µL appropriate complete culture medium and incubated for 24 h to allow cells to attach. Cells were then treated for 72 h, then WST1 (10 µL) reagent (Roche, New York, NY, USA) was added to each well according to the manufacturer’s instructions. After 4 h of the recommended incubation time, the absorbance of each sample was measured using a microplate (ELISA) reader at 450 nm. The absorbance value of the blank wells (containing only medium and WST-1 reagent) was subtracted from the absorbance of each sample, and then the values of treated samples were normalized to the controls. Control samples were represented as 100% cell viability, and the percentage of viable cells in treated samples was calculated relative to the controls. These experiments were performed in triplicate and repeated 3 times.

### 2.4. Apoptosis Analysis by Annexin V/Flow Cytometry

Cells were harvested, centrifuged, and stained with propidium iodide (PI) and Alexa Flour 488 annexin V (Invitrogen, Carlsbad, CA, USA), and then were analyzed by flow cytometry.

### 2.5. RNA Purification and qRT-PCR

mRNeasy mini kit (Qiagen, Manchester, UK) was used to purify total RNA as recommended by the manufacturer’s instructions. RNA concentration and integrity were determined using Agilent 2100 bioanalyzer system and associated Agilent 2100 expert software. RNA (1 µg) was used to synthesize complementary deoxyribonucleic acid (cDNA) using the Advantage RT-PCR kit (Clontech Laboratories, Mountain View, CA, USA) following the manufacturer’s instructions. Amplifications were performed using FastStart Essential DNA Green Master (Roche) and were performed using the LightCycler^®^ 96 Real-time PCR detection system (Roche) according to the following cycle conditions: 95 °C for 10 min (1 cycle); 95 °C for 10 s, 59 °C for 20 s, and 72 °C for 30 s (45 cycles). GAPDH was used for normalization, and gene expression differences were calculated using the threshold cycle (Ct) by LightCycler^®^ 96 SW 1.1 software. These experiments were performed in triplicate and repeated 3 times. The primer sequences are:
PPRC1: Forward: 5’-TCTCCCCATCCGAAACACAA-3’ 
Reverse: 5’-GTCTCTTGGGTCTCAGGCTT-3’c-MYC: Forward: 5’-CCTGGTGCTCCATGAGGAGAC-3’
Reverse: 5’-CAGACTCTCCAGCATCCACT-3’GAPDH: Forward: 5’-GAG TCC ACT GGC GTC TTG-3’
Reverse: 5’-GGG GTG CTA AGC AGT TGG T-3’

### 2.6. Cellular Lysate Preparation and Immunoblotting

Cells were harvested and centrifuged, and the pellets were homogenized with RIPA buffer (Sigma-Aldrich, St. Louis, MI, USA) supplemented with protease inhibitors (Roche). Cells were incubated on ice with a brief vortex every 10 min for 2 h. The obtained cell lysates were centrifuged, and the resulting supernatants were stored at −80 °C. Extracted proteins (50 μg) were separated using SDS-PAGE and then were transferred to polyvinylidene difluoride membrane (PVDF) (Bio-Rad), which was first blocked with 5% powdered skimmed milk in Tris-buffered saline with tween (TBST) (Sigma-Aldrich) for 1 h, then was incubated with the appropriate primary antibody (diluted in TBST as recommended by the suppliers) overnight, and then with an appropriate secondary antibody (Promega, Southampton, UK) for 1 h. As negative controls, membranes were stained only with secondary antibodies. Visualization of the secondary antibody was performed using a chemiluminescence detection procedure according to the manufacturer’s protocol (Thermo Fisher Scientific, Waltham, MA, USA).

Primary antibodies directed against cleaved-PARP [Asp214] (cat#9541), cleaved caspase-9 [Asp315] (cat#9505), cleaved caspase-3 [Asp175] (cat#9664) were purchased from Cell Signaling Technology. These experiments were repeated 3 times.

### 2.7. Quantification of Protein Expression Level

The protein signal intensity of each band was determined using ImageJ version 1.67. Next, dividing the obtained value of each band by the value of the corresponding internal control allowed a correction of the loading differences. The fold change in the protein levels was determined by dividing the corrected values by that of the control.

### 2.8. Cell Proliferation

These assays were carried out using xCELLigence Real-Time Cell Analysis (RTCA) as recommended by the manufacturer (ACEA Biosciences, Santa Clara, CA, USA). Cells (5 × 10^3^–2 × 10^4^) were seeded in E-plates in complete medium and then incubated for 72 h. The RTCA (1.2.1) software was used to analyze the obtained results expressed as Cell Index (CI) values. These experiments were performed in triplicate and were repeated at least 3 times.

### 2.9. Mitochondrial Staining

Mitochondrial staining was performed using MitoTracker Red CMXRos (Invitrogen, Carlsbad, CA, USA); cells were cultured on coverslip at 10^6^ per mL loaded with 1 µM Mito for 5 min at 37 °C, washed twice with PBS, and viewed under the Yokogawa Spinning Disk confocal microscopy system (Carl Zeiss, Cambridge, UK).

### 2.10. Data Mining

RNA expression data, along with clinicopathological characteristics of the patients, were downloaded for different cancer types using The Human Protein Atlas (https://www.proteinatlas.org/) database [18], accessed on 15 March 2018. Patients were divided into “high” and “low” expression categories based on the median value of PPRC1 expression. The database also contains more than 60 cell lines representing various cell populations from different human organs and tissues and gives expression values for around 12,073 genes. PPRC1 expression values were obtained from different cancer cell lines. PPRC1 expression was compared between the cancer and the control datasets using Expression Atlas at European Bioinformatics Institute (http://www.ebi.ac.uk/gxa), accessed on 17 March 2018. The log values of expression for the cancer and normal were plotted using a heat map. Mutations and copy number alterations for PPRC1 gene were retrieved using open access database cBioPortal (http://www.cbioportal.org), accessed on 20 March 2018, which currently holds more than 80,000 tumor samples from 242 cancer studies in the Cancer Genome Atlas (TCGA) pipeline [19,20]. The clinicopathological characteristics of the patients with primary focus on age, gender, stage, disease-free survival (DFS), and overall survival (OS) in months available in the database were also retrieved.

### 2.11. Mutation Pathogenicity and Data Visualization

All the missense mutations were processed using three different prediction algorithms (SIFT, PolyPhen, and CADD) [13,21,22]. A mutation was deemed as likely pathogenic if it was predicted as truncating by at least two of the three computational algorithms. All the frameshift, nonsense, and splicing mutations (±1 or 2) were considered as likely pathogenic. Mutation data were plotted using lollipop plots from Mutation Mapper (version 1.2.0) showing the type and frequency of mutations.

### 2.12. Statistical Analysis

Statistical analysis was performed using a two-tailed unpaired Student’s *t*-test. *p* values of 0.05 and less were considered statistically significant. These analyses were performed using the GraphPad Prism software (Version 9).

Statistical analysis was performed using the Survival package in R version 3.5. Survival analyses were performed by using the Kaplan–Meier method. The Kaplan–Meier curves were generated to evaluate the association between the mutation, copy number variations, and expression level of PPRC1 and patient survival. Univariate and multivariate Cox proportional hazard regression analyses were used to identify independent predictors of survival. Statistical significance was defined as *p* < 0.05.

## 3. Results

### 3.1. Leukemia Cell Lines and Primary Cells Are Sensitive to Eugenol

**Figure 1:**
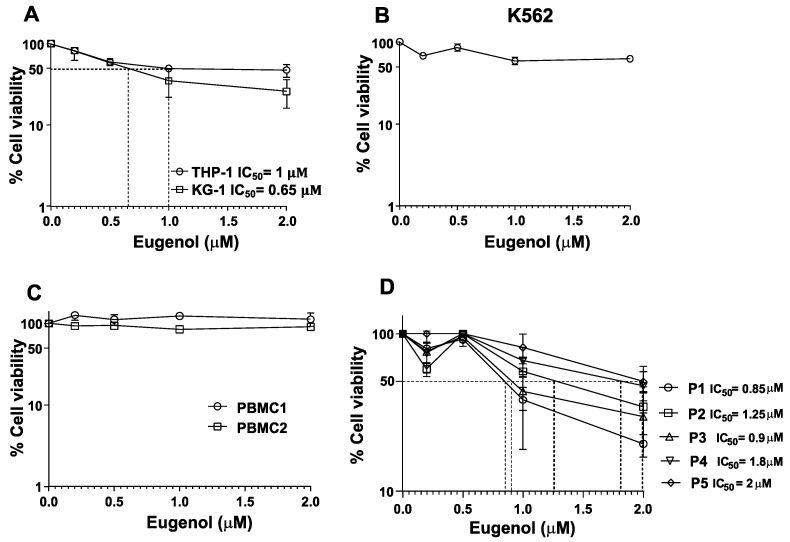
Cytotoxic effects of eugenol on leukemia cells. (**A**,**B**) Exponentially growing cells were challenged with the indicated concentrations of eugenol for 72 h, and then cell viability was assessed by the WST1 assay. (**C**,**D**) PBMCs were isolated from healthy donors (**C**) or from AML patients (**D**), and then were treated as explained above. Error bars represent mean ± SEM (n = 3).

Exponentially growing leukemia cells (THP-1 and KG-1) were either sham-treated (ethanol) or exposed to eugenol (0.2, 0.5, 1, and 2 μM) for 3 days, and then the cytotoxic effect of eugenol was assessed using the WST-1 assay. Figure 1A shows a strong cytotoxic effect on both cell lines THP-1 (IC_50_ = 1 μM) and KG-1 (IC_50_ = 0.65 μM). However, the CML cell line (K-562) and PBMCs isolated from two healthy donors exhibited only marginal cytotoxicity in response to the same doses of eugenol (Figure 1B,C). Interestingly, PBMCs isolated from five AML patients also showed sensitivity to the same doses of eugenol with different IC_50_ (0.85, 0.9, 1.25, 1.8, and 2 μM) (Figure 1D). This shows that eugenol possesses cytotoxic effects against AML cell lines and PBMCs from AML patients but not from healthy donors.

### 3.2. Eugenol Promotes Apoptosis and Inhibits Proliferation in AML Cells

**Figure 2:**
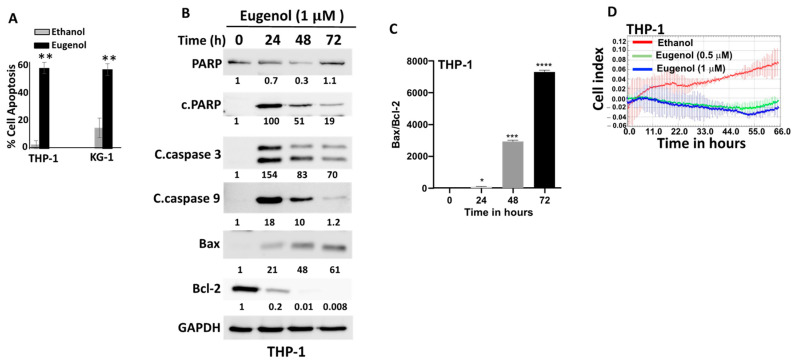
Eugenol promotes apoptosis and inhibits proliferation in AML cells. (**A**) Cells were either sham-treated (ethanol) or challenged with the indicated doses of eugenol for 72 h, and then the proportions of apoptosis were analyzed by Annexin V/PI-flow cytometry and were presented as a histogram. Error bars represent mean ± SEM (n = 3). ** *p* ≤ 0.01. (**B**) Cells were treated with eugenol (1 μM) for the indicated periods of time, and then cell lysates were prepared and used for immunoblotting analysis using specific antibodies for the indicated proteins. The numbers below each band represent fold changes relative to the control (0) after normalization to GAPDH. (**C**) The histogram shows Bax/Bcl-2 ratio of the values determined in (**B**). Error bars represent mean ± SEM (n = 3). * *p* ≤ 0.05, *** *p* ≤ 0.001, **** *p* ≤ 0.0001. (**D**) Cells (10^4^) were treated as indicated and were seeded in the E-plate in the presence of complete medium, and then cell proliferation capacity was assessed using the RTCA-DP xCELLigence System. Data are representative of different experiments performed in triplicate.

In order to confirm the cytotoxic effect of eugenol on AML cells and determine the killing pathway, we decided to make use of the annexinV/PI-flow cytometry technique. To this end, cells (THP-1 and KG-1) were either sham-treated (ethanol) or challenged with eugenol (1 μM) for 72 h and then stained with annexinV/PI. Figure 2A shows eugenol-dependent induction of apoptosis reaching 60% in both cell lines. To confirm this, THP1 cells were treated with eugenol (1 μM) for different periods of time (0, 24, 48, and 72 h), and then whole-cell lyses were prepared and analyzed by immunoblotting using specific antibodies against apoptotic proteins, while GAPDH was utilized as an internal control. Figure 2B shows an eugenol-dependent decrease in the level of PARP and a strong increase (100-fold) in the level of the active form of the protein (cleaved-PARP). Similarly, the levels of cleaved caspase-3 and cleaved caspase-9 were also increased (Figure 2B). This shows eugenol-dependent induction of apoptosis through the intrinsic mitochondrial pathway. This was confirmed by showing a sharp time-dependent decrease in the level of the anti-apoptosis protein Bcl-2 and an increase in the level of the pro-apoptosis protein Bax (Figure 2B). This led to a huge and time-dependent increase in the level of the Bax/Bcl-2 ratio (Figure 2C).

To assess the anti-proliferative effect of eugenol on AML cancer cells, THP-1 cells were treated either with eugenol (0.5 and 1 μM) or with ethanol used as a negative control. Cell proliferation was measured over 72 h using the xCELLigence real-time cell analyzer (RTCA). Figure 2D shows that eugenol abolished the cell proliferation ability of THP-1 cells as compared to controls. This indicates that eugenol has a strong inhibitory effect on the proliferation of AML cells in vitro.

### 3.3. Eugenol Promotes Apoptosis and Inhibits Proliferation in Cytarabine-Resistant AML Cells

**Figure 3:**
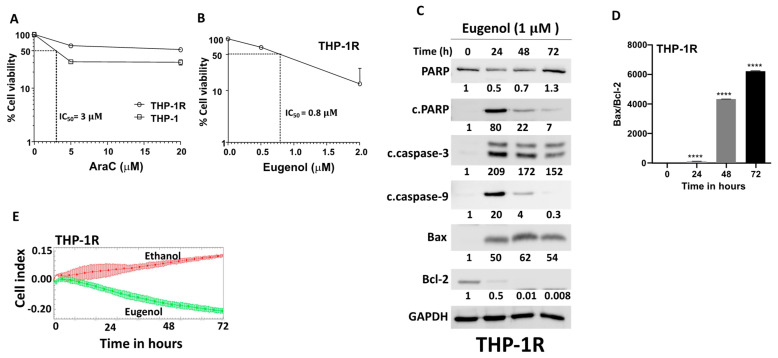
Eugenol promotes apoptosis and inhibits proliferation in cytarabine-resistant AML cells. (**A**,**B**) Exponentially growing cells (THP-1 and the AraC-resistant cells: THP-1R) challenged with the indicated concentrations of eugenol for 72 h, and then cell viability was assessed by the WST1 assay. (**C**) Cells were treated with eugenol (1 μM) for the indicated periods of time, and then cell lysates were prepared and used for immunoblotting analysis using specific antibodies for the indicated proteins. The numbers underneath each band represent fold changes relative to the control (0) after normalization to GAPDH. (**D**) The histogram shows Bax/Bcl-2 ratio. Error bars represent mean ± SEM (n = 3). **** *p* ≤ 0.0001. (**E**) Cells (10^4^) were treated as indicated and were seeded in the E-plate in the presence of complete medium, and then cell proliferation capacity was assessed using the RTCA-DP xCELLigence System. Data are representative of different experiments performed in triplicate.

Cytarabine (AraC), the widely used and most effective single agent for the treatment of AML patients, generates a high remission rate. However, a great proportion of these patients develop resistance and relapse [6]. To address this vital issue, we decided to develop AraC-resistant THP-1 cells (THP-1R) and test the cytotoxic effects of eugenol. THP-1R cells were developed from THP-1 cells that resisted the cytotoxic effect of cytarabine used at 2 μM. Figure 3A shows the high resistance to AraC of THP-1R cells relative to their corresponding original THP-1 cells. However, THP-1R cells were sensitive to eugenol with a low IC_50_ (0.8 μM) (Figure 3B). This sensitivity of THP-1R cells to eugenol was confirmed by showing eugenol-dependent induction of apoptosis in these cells (Figure 3C). Indeed, eugenol upregulated the levels of cleaved PARP, cleaved caspase-3, and cleaved caspace-9 to levels similar to those observed in THP-1 cells (Figure 3C). Interestingly, eugenol strongly downregulated Bcl-2 and upregulated Bax, which generated a strong increase in the Bax/Bcl-2 ratio (Figure 3D). This shows that eugenol promotes apoptosis through the mitochondrial pathway in the THP-1R cells as well. In addition, eugenol suppressed the proliferative capacity of THP-1R cells (Figure 3E).

### 3.4. Eugenol Downregulates c-MYC and PPRC1 in AML Cell Lines

**Figure 4:**
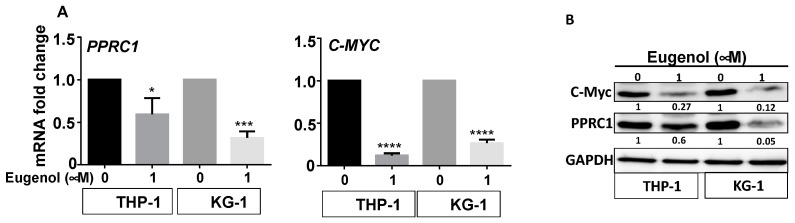
Eugenol downregulates *c-MYC* and *PPRC1* in AML cell lines. Cells were either sham-treated or exposed to eugenol for 24 h. (**A**) Total RNA was extracted and the mRNA levels of the indicated genes were assessed by qRT-PCR. Error bars represent mean ± SEM (n = 3). * *p* ≤ 0.05, *** *p* ≤ 0.001, **** *p* ≤ 0.0001. (**B**) Whole-cell lysates were prepared and used for immunoblotting analysis. The numbers below each band represent fold changes relative to the control (0) after normalization to GAPDH.

In order to delineate the effects of eugenol on leukemia cells at the molecular level, we sought to perform RNAseq experiments on sham-treated and eugenol-challenged (1 μM) cells. Among the genes that showed high differential expression, *c-MYC* and its downstream target *PPRC1*. This was confirmed by qRT-PCR and immunoblotting in two cell lines (THP-1 and KG-1) (Figure 4A,B). Indeed, eugenol reduced the expression of both genes at the mRNA and protein levels in both cell lines, but the effect was stronger in KG-1 cells (Figure 4A,B). This indicates that eugenol can inhibit the expression of these two genes at the mRNA level.

### 3.5. Eugenol Reduces Mitochondrial Membrane Potential (MMP)

**Figure 5:**
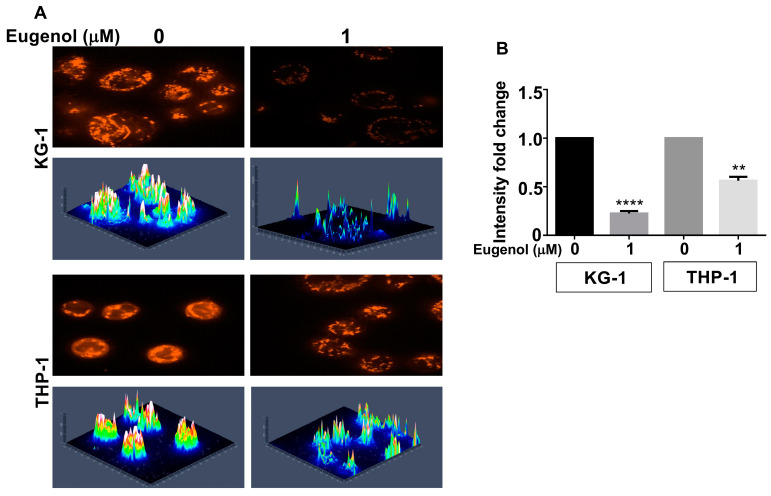
Eugenol reduces mitochondrial membrane potential (MMP). Cells were either sham-treated (ethanol) or challenged with eugenol (1 μM) for 24 h and then were labeled with MitoTracker Red CMXRos (1 µM). (**A**) Cells were photographed using confocal fluorescence microscope. Three-dimensional intensity was determined. (**B**) Histogram showing intensity fold change as determined in (**A**); error bars represent mean ± SEM (n = 3). ** *p* ≤ 0.01, **** *p* ≤ 0.0001.

Since PPRC1 is known to play important roles in mitochondrial biogenesis, we decided to test the effect of eugenol on mitochondria in two cell lines, THP1 and KG-1, using MitoTracker Red CMXRos. Compared with mitochondria in control cells, the mitochondria in eugenol-treated cells exhibited aberrant morphology and a less MitoTracker Red CMXRos fluorescence pattern (Figure 5A). The three-dimensional intensity maps show lower intensity in treated cells compared to control (4-folds for KG1) and (1.5-folds for THP1) (Figure 5B). This indicates the presence of a lower number of healthier mitochondria with physiological membrane potential (PMM) in eugenol-treated cells compared to controls.

### 3.6. PPRC1 Expression Is Higher in Cancer Cells Relative to Normal Cells and Significantly Correlates with Short Overall Survival in Several Cancers

**Figure 6:**
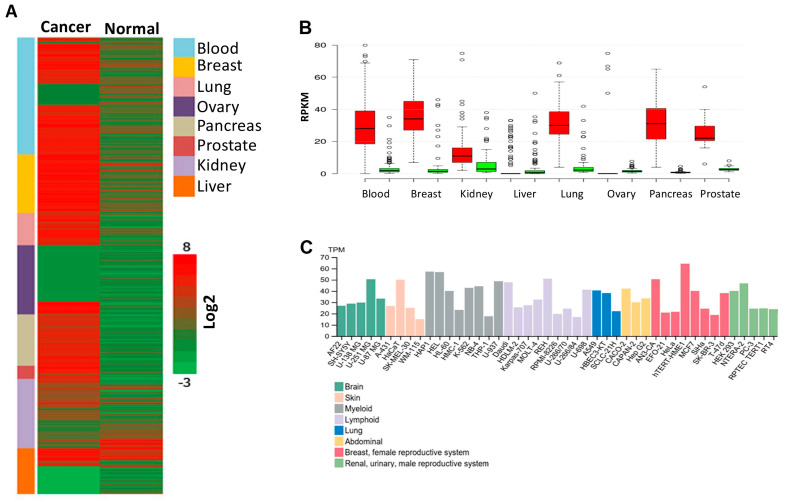
*PPRC1* expression is higher in cancer cells and significantly correlates with short overall survival. (**A**) mRNA expression of *PPRC1* in cancer cases versus their normal counterparts was plotted using a heat map. (**B**) *PPRC1* mRNA expressions (from Figure 1A) are significantly higher (*p* < 0.001) in most cancer types (red color) as compared to their normal counterparts (green color). (**C**) Basal *PPRC1* mRNA expression in different cancer cell lines.

Next, we investigated the expression level of PPRC1 in cancer versus normal cells. To this end, we made use of publicly available gene expression data sets from EMBL-EBI Expression Atlas (https://www.ebi.ac.uk/gxa/home, accessed on 17 March 2018) and The Genotype-Tissue Expression (GTEx) (https://gtexportal.org, accessed on 20 March 2018), which contain RNA-seq data from cancer cases and their normal counterparts. Our extensive analysis of 1330 cancer and normal cases has shown that the *PPRC1* expression was higher compared to normal tissues (Figure 6A). Similarly, a comparison of *PPRC1* expression levels in cancer versus normal cells in different cancer types showed that *PPRC1* expression was significantly higher (*p* < 0.001) in primary cells from the various cancer types tested (Figure 6B). In addition, we also studied the expression level of PPRC1 in cancer cell lines from multiple origins using Human Protein Atlas datasets available at (www.proteinatlas.org, accessed on 15 March 2018) [18]. We have found PPRC1 to be highly expressed in most cancer cell lines (Figure 6C). These findings from tumors’ primary cells, as well as cancer cell lines, suggest that PPRC1 expression levels are higher in neoplastic relative to normal cells, which might suggest an oncogenic function of the *PPRC1* gene.

**Figure 7:**
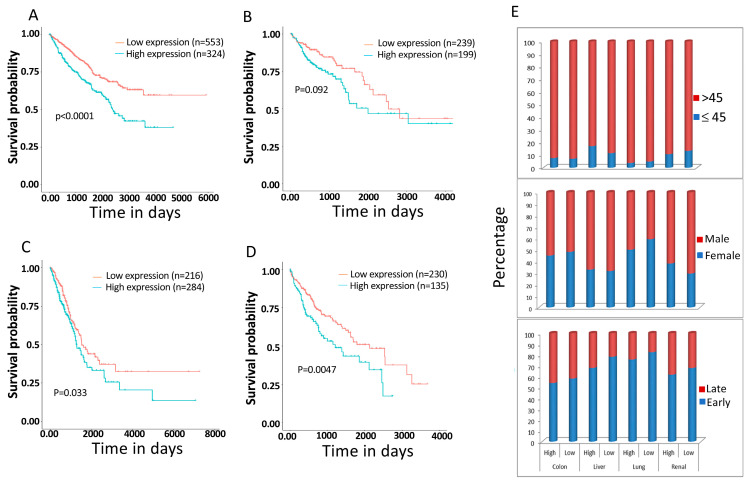
High PPRC1 expression correlates significantly with short overall survival. Kaplan–Meier curves for correlation of high and low expression of PPRC1 (**A**) Renal cancer patients. (**B**) Colon adenocarcinoma patients. (**C**) Lung adenocarcinoma patients. (**D**) Liver cancer patients. (**E**) Correlation of high/low expression of PPRC1 with clinical parameters, including tumor stages, age, and sex.

Since primary tumor cells and cancer cell lines express high levels of PPRC1, we sought to investigate the possible correlation between the expression level of the gene and clinical outcomes in cancer patients. To achieve this, we have utilized the RNA seq datasets from the Protein Atlas database, which was generated by The Cancer Genome Atlas (TCGA) [18]. We have found that high *PPRC1* expression significantly correlates with short overall survival (OS) in renal cancer (*p* < 0.0001; 877 patients), colon adenocarcinoma (*p* < 0.0092; 438 patients), lung adenocarcinoma (*p* < 0.033; 500 patients), and liver cancer (*p* < 0.0043; 365 patients) (Figure 7A–D). On the other hand, our analysis shows that PPRC1 expression is independent of age, gender, and tumor stages (Figure 7E).

### 3.7. Genetic Alterations in the PPRC1 Gene Correlate Significantly with Severe Clinical Outcome in Cancer Patients

**Figure 8:**
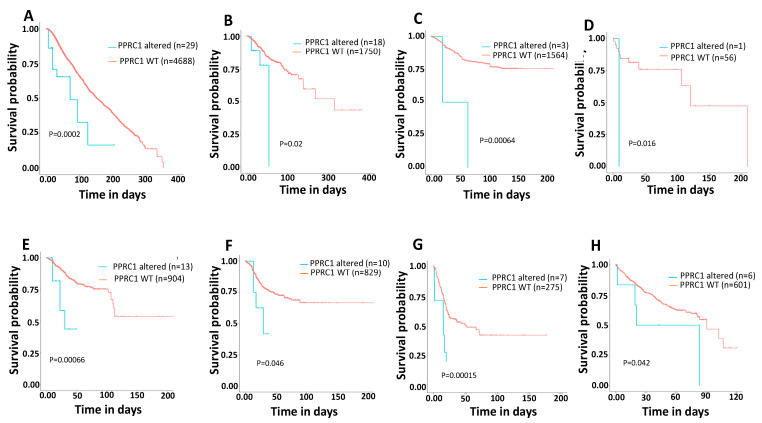
Genetic alterations in PPRC1 correlate significantly with short OS and DFS in several cancer types. Kaplan–Meier survival curves for correlation of PPRC1 wild-type gene versus mutated. (**A**) Overall survival in breast (*p* = 0.0002). (**B**) Disease-free survival in breast (*p* = 0.02). (**C**) Overall survival in Lymphoma (*p* = 0.0006). (**D**) Disease-free survival in Lymphoma (*p* = 0.016). (**E**) Overall survival in Uterus (*p* = 0.0006). (**F**) Disease-free survival in Uterus (*p* = 0.0452). (**G**) Overall survival in Pancreas (*p* = 0.0001). (**H**) OS in Liver (*p* = 0.01). Number of wild-type and altered PPRC1 cases for each cancer are shown in each panel.

Next, we tested the correlation between genetic alterations in the *PPRC1* gene and the patient’s clinical outcome. To address this, we utilized the cBioPortal for Cancer Genomics, which contains large-scale cancer genomics data sets [19,20]. We have found that the *PPRC1* gene alterations correlate significantly with both short OS and Disease-Free Survival (DFS) in breast cancer (*p* = 0.0002 and *p* = 0.02, respectively) (Figure 8A,B), Lymphoma (*p* = 0.0006 and *p* = 0.016, respectively) (Figure 8C,D), Uterus (*p* = 0.0006 and *p* = 0.0452, respectively) (copy number mutations only) (Figure 8E,F) and short OS in Pancreas (*p* = 0.0001) (Figure 8G) and Liver (*p* = 0.01) (Figure 8H). Thus, these findings indicate the role of PPRC1 mutations in aggressive clinical outcomes. However, correlations of PPRC1 genetic alterations with clinicopathological characteristics, such as tumor stage, sex, and age, have shown no substantial difference.

**Figure 9:**
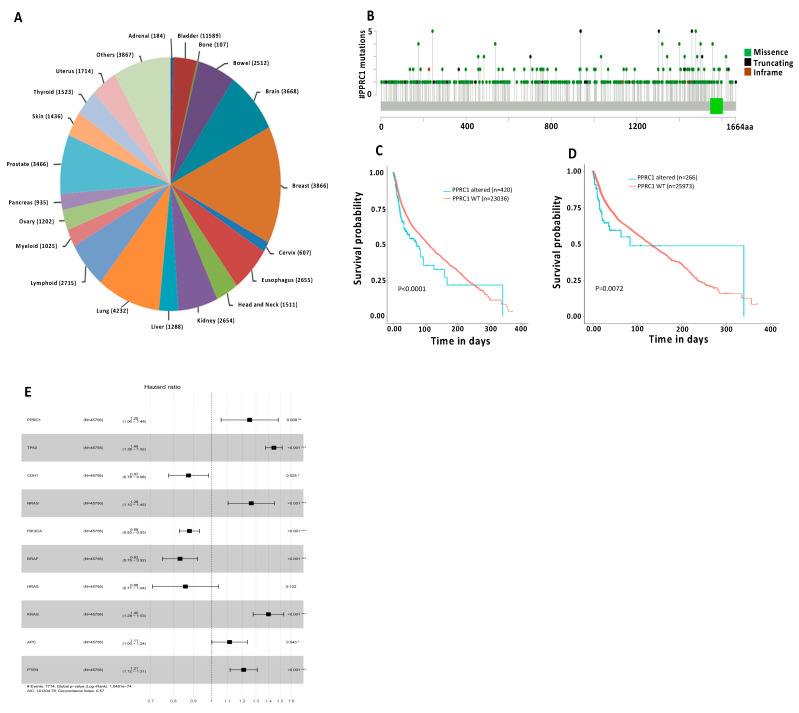
Impact of PPRC1 gene alterations on overall survival in very large cancer patient’s cohort. (**A**) Number, percentage, and type of cancer patients used in the analysis. (**B**) Mutation mapper of the PPRC1 gene. Locations of missense, in-frame, and truncating mutations were shown. (**C**) Kaplan–Meier survival curves for correlation of PPRC1 wild-type gene versus mutated in all reported cancer patients (*p* < 0.0001). (**D**) Kaplan–Meier survival curves for correlation of PPRC1 wild-type gene versus mutated in all reported cancer patients, excluding cases carrying mutations in TP53, PICK3CA, and PTEN (*p* = 0.009). (**E**) Multivariate analysis of PPRC1 and additional other key tumor suppressors/oncogenes. * *p* ≤ 0.05, ** *p* ≤ 0.01, *** *p* ≤ 0.001.

We have also found that the mutation frequency of the *PPRC1* gene was 1.43% in our 45,756 patient cohort. The number and percentage of each cancer type used in this analysis are summarized in Figure 9A. The type, location, and percentage of each mutation in the PPRC1 gene are shown in Figure 9B. Since out of 45,756 cancer patients, only 23,456 patients have survival data, we went further to test whether PPRC1 alterations correlate with short OS in those patients with different cancer types. Interestingly, our analysis indicates that PPRC1 alterations were significantly correlated with short OS (*p* < 0.0001) in this large cohort (Figure 9C).

To test whether the prognostic power of PPRC1 is driven by the association with key tumor suppressors/oncogenes, such as TP53, PIK3CA, and PTEN, we have removed from our patient cohort all cancer patients (9919 cases) that carry these genetic alterations and re-analyzed the data with respect to the correlation of PPRC1 genetic alterations with overall survival. Our analysis shows that PPRC1 alterations are still significantly correlated with short overall survival (*p* = 0.0072) in the absence of patients carrying mutations in TP53, PIK3CA, and PTEN (Figure 9D). Next, we have analyzed whether PPRC1 can provide independent prognostic value to additional key tumor suppressors/oncogenes. Interestingly, our multivariate analysis showed that the prognostic value of PPRC1 mutation is significantly independent (*p* = 0.009) of TP53, PIK3CA, PTEN, NRAS, HRAS, KRAS, BRAF, APC, and CDH1 (Figure 9E). Moreover, we have identified specific mutations present in multiple cancer types, such as the *P941Tsf* mutation, which is present in nine different types of cancer (Table 1). Only mutations that were found in four or more cancer types are listed in Table 1. Interestingly, three mutations at proline present in positions 938, 940, and 941 were identified in eleven types of cancer, which suggests that proline at these positions could be critical for the function of the PPRC1 protein.

## 4. Discussion

In the present report, we present clear evidence that eugenol, a natural essential oil with recognized anti-cancer properties [15,16,17], has potent and specific anti-carcinogenic effects against AML cell lines and primary AML cells. Indeed, AML cells are sensitive to eugenol, which promotes apoptosis through the intrinsic mitochondrial pathway and inhibits their proliferative potential. Interestingly, these effects were also observed in cytarabine-resistant cells. In both cases, eugenol had a very strong time-dependent upregulation of Bax and downregulation of the ant-apoptotic protein Bcl-2, which became undetectable after 72 h of treatment. This led to very high Bax/Bcl-2 ratios (Figure 2B and Figure 3C). It is well known that anti-apoptotic proteins such as Bcl-2 play key roles in the survival of AML cells and poor prognosis of the patients [23,24,25]. Several Bcl-2 inhibitors were tested over the last two decades, and venetoclax is currently in use as an anti-AML treatment molecule [26]. This suggests that the use of eugenol as a potent inhibitor of Bcl-2 could be of great therapeutic value, especially for those who show resistance to venetoclax or cytarabine. Resistance to cytarabine, the primary component of AML therapy, is indeed a well-known phenomenon among AML patients [7]. Thereby, eugenol can help in targeting these cytarabine-resistant cells in AML patients and improve their therapeutic outcomes through Bcl-2 downregulation. Eugenol may also sensitize these cells to chemotherapeutic agents such as anthracyclines. Indeed, we have shown that cytarabine-resistant AML cells are sensitive to the killing effect of eugenol, which inhibits their proliferative capacity as well. In addition to Bcl-2, eugenol targeted two other mitochondrial metabolism-related proteins, namely PPRC1 and its upstream regulator c-Myc, which are involved in mitochondrial biogenesis [27]. Indeed, eugenol strongly downregulated *PPRC1* and *c-MYC* and reduced mitochondrial membrane potential. This indicates that the anti-AML effect of eugenol could be mediated by targeting these two genes. *c-MYC* is a well-known oncogene and is one of the most commonly activated in human cancers, including leukemia [28]. Indeed, c-Myc is frequently activated in AML, in particular by mutations in the Flt3 receptor tyrosine kinase, the most prevalent type of mutations in AML [29,30]. However, the implication for PPRC1 regulation still remains elusive. It is also possible that the eugenol-dependent regulation of PPRC1 is indirect through other regulatory pathways. These important in vitro findings need to be confirmed in vivo in an adequate AML animal model.

In order to shed light on the role of PPRC1 in carcinogenesis, a comprehensive analysis of transcriptomic and genomic databases was performed and demonstrated the following: (1) PPRC1 expression is significantly higher in most cancer types tested, (2) high PPRC1 expression was correlated with short OS in multiple cancers, (3) PPRC1 gene alterations significantly correlated with short OS and/or DFS of several types of cancer, (4) PPRC1 prognostic power is independent of mutations in well-known tumor suppressors/oncogenes. We have shown that the expression of PPRC1 is significantly higher in a substantial number of renal cancer, colon adenocarcinoma, lung adenocarcinoma, blood, breast, and liver cancer cases. However, unlike the transcriptome data, PPRC1 mutations, including gene amplification, are very low in breast, lymphoma, uterus, pancreas, and liver cases. This suggests that there are additional post-transcriptional modifications that lead to the increase in the PPRC1 level in tumors.

Furthermore, we have also tested whether the prognostic power of PPRC1 could still be valid if it is applied to a much larger patient cohort. Importantly, in a very large cohort of 23,456 cancer patients, PPRC1 alterations were significantly correlated with short OS (*p* < 0.0001). Next, we excluded 9919 patients carrying mutations in TP53, PIK3CA, and PTEN from our cohort to test whether the correlation between PPRC1 and poor survival is associated with these well-known tumor suppressors/oncogenes. Strikingly, PPRC1 mutations showed an adverse impact in the absence of TP53, PIK3CA, and PTEN mutations. Interestingly, our multivariate analysis showed that PPRC1 mutations are independent of mutations in key tumor suppressors/oncogenes, including TP53, PIK3CA, PTEN, NRAS, HRAS, KRAS, BRAF, APC, and CDH1. Moreover, we report that the *P941* mutation is present in nine different types of cancer. In fact, mutations at proline positions 938, 940, and 941 were found in eleven different cancer types, which suggests that disrupting these amino acid residues might activate the tumorigenesis function of PPRC1. Our analysis also shows that PPRC1 mutations include amplification, missense, and nonsense mutations. This suggests that the various types of PPRC1 mutations may lead to the stabilization of the protein and/or an increase in its activity. Therefore, there is a need to functionally characterize these mutations in order to understand how each mutation type can affect the activity of the *PPRC1* gene.

PPRC1 has exhibited some oncogenic characteristics, which parallel some known functions of the gene. Indeed, it has been shown that *PPRC1* silencing causes cell cycle arrest and blocks proliferation of U2OS cells [12]. Furthermore, it has been reported that *PPRC1* up-regulation is mediated by the c-Myc/AKT signaling in response to mitochondrial stress. This activation can be triggered during tumorigenesis [31]. In addition, the *PPRC1* mRNA has a short half-life and is lowly expressed in quiescent cells, but the *PPRC1* mRNA level rapidly increases upon serum stimulation, indicating a proto-oncogene property [32]. In thyroid cancer cell lines, PGC-1-related genes were found to promote respiratory chain machinery and increase mitochondrial mass [33,34]. Taken together, these findings indicate that PPRC1 may have an oncogenic function in various types of tumors. Thereby, PPRC1 could be considered a promising therapeutic target not only for AML patients but also for other types of cancer.

## 5. Conclusions

The present findings indicate that eugenol has potent anti-AML effects by targeting c-MYC and its downstream effector, PPRC1. This key player in mitochondrial biogenesis plays oncogenic functions in various types of cancer. Further mechanistic studies are needed to fully reveal the role of this gene in cancer biology and its potential use for novel targeted therapeutic approaches.

## Figures and Tables

**Table 1:** List of most frequent PPRC1 mutations found in different cancer types. Only a mutation in four or more different cancers is listed.

Mutation Name	Mutation Type	Cancer Type
P941Tfs	Frameshift insertion	Stomach Adenocarcinoma
P941Tfs	Frameshift insertion	Renal Clear Cell Carcinoma
P941Tfs	Frameshift insertion	Prostate Adenocarcinoma
P941Tfs	Frameshift insertion	Breast Invasive Ductal Carcinoma
P941Tfs	Frameshift insertion	Colorectal Adenocarcinoma
P941Tfs	Frameshift insertion	Small Cell Lung Cancer
P941Tfs	Frameshift insertion	Glioblastoma
P941fs	Frameshift deletion	HEC151 cell line, Endometrial adenocarcinoma
P941fs	Frameshift deletion	HEC6 cell line, Endometrial endometrioid adenocarcinoma
P941fs	Frameshift deletion	CCK81 cell line, Colon adenocarcinoma
P941fs	Frameshift deletion	SNU-1 cell line, Gastric carcinoma
P938fs	Frameshift insertion	22RV1 cell line, Prostate carcinoma
P938fs	Frameshift insertion	HEC-265 cell line, Endometrial adenocarcinoma
P938fs	Frameshift insertion	MDAPCA2B cell line, Prostate carcinoma
P938fs	Frameshift insertion	SNU407 cell line, Colon adenocarcinoma
P938fs	Frameshift insertion	CW-2 cell line, Colon adenocarcinoma
P938fs	Frameshift insertion	EFO27 cell line, Ovarian mucinous adenocarcinoma
R243H/C	Missense	Prostate Adenocarcinoma
		Lung Squamous Cell Carcinoma
		Uveal Melanoma
		Uterine Endometrioid Carcinoma
		Colon Adenocarcinoma
P940Hfs	Frameshift deletion	Prostate Adenocarcinoma
		Stomach Adenocarcinoma
		Uterine Endometrioid Carcinoma
		Colorectal Adenocarcinoma
R177C	Missense	Uterine Endometrioid Carcinoma
		Colon Adenocarcinoma
		Bladder Urothelial Carcinoma
		Stomach Adenocarcinoma
P537S/L/T	Missense	Cutaneous Squamous Cell Carcinoma
		Cutaneous Melanoma
		Melanoma
		Hepatocellular Adenoma
R1320*/P/Q	Nonsense/Missense	Lung Adenocarcinoma
		Cutaneous Melanoma
		Bladder Urothelial Carcinoma
		Cutaneous Squamous Cell Carcinoma
R1400Q/L/W	Missense	Adenoid Cystic Carcinoma
		Colorectal Adenocarcinoma
		Serous Ovarian Cancer
		Cutaneous Melanoma
R1400P		KCL22 cell line, Chronic myelogenous leukemia
R1556Q/*	Missense/Nonsense	Pancreatic Adenocarcinoma
		Uterine Endometrioid Carcinoma
		Colorectal Adenocarcinoma
		Cutaneous Melanoma

## Data Availability

The datasets used and/or analyzed during the current study are available from the corresponding author upon reasonable request.

## Abbreviations

The following abbreviations are used in this manuscript:

MMP	Mitochondrial membrane potential
PBMCs	Peripheral Blood Mononuclear cells
AML	Acute myeloid leukemia
DFS	Disease-free survival
OS	Overall survival
